# A T2 Translational Science Modified Delphi Study: The Ethical Triage and Treatment of Entrapped and Mangled Extremities in Resource‐Scarce Environments

**DOI:** 10.1002/wjs.12486

**Published:** 2025-02-20

**Authors:** Eric S. Weinstein, Zachary Gilbert, James Gosney, Brielle Weinstein, Hannah B. Wild, Joseph Cuthbertson, Melissa Leming, Rachel Semmons, Dónal O'Mathúna, Carl Montan, Richard Gosselin, Frederick “Skip” Burkle

**Affiliations:** ^1^ Department of Emergency Medicine—Morsani College of Medicine University of South Florida Tampa Florida USA; ^2^ University of South Florida Health Center for Advanced Medical Learning and Simulation (CAMLS) Tampa Florida USA; ^3^ CRIMEDIM ‐ Center for Research and Training in Disaster Medicine, Humanitarian Aid and Global Health Università del Piemonte Orientale Novara Italy; ^4^ Emergency Rehabilitation Committee International Society of Physical and Rehabilitation Medicine Milan Italy; ^5^ Department of Plastic Surgery Morsani College of Medicine University of South Florida Tampa Florida USA; ^6^ Department of Surgery, University of Washington Seattle Washington USA; ^7^ Explosive Weapons Trauma Care Collective International Blast Injury Research Network, University of Southampton Southampton UK; ^8^ Monash University Disaster Resilience Initiative Monash University Monash Australia; ^9^ School of Medicine University of Notre Dame Australia Fremantle Western Australia Australia; ^10^ College of Nursing and the Center for Bioethics and Medical Humanities The Ohio State University Columbus Ohio USA; ^11^ Department of Molecular Medicine and Surgery Karolinska University Hospital Karolinska Institutet Stockholm Sweden; ^12^ Orthopedic Surgery Institute for Global Orthopaedics and Traumatology University of California at San Francisco El Granada California USA; ^13^ Global Scholar Woodrow Wilson International Center for Scholars Washington DC USA

**Keywords:** clinical practice guidelines, Delphi study, entrapped extremity, mangled extremity, resource‐scarce environment, translational science

## Abstract

**Background:**

There is a lack of ethical triage and treatment guidelines for the entrapped and mangled extremity (E&ME) in resource‐scarce environments (RSE): mass casualty incidents, low‐ to middle‐income countries, complex humanitarian emergencies including conflict, and prolonged transport times (RSE). The aim of this study is to use a modified Delphi (mD) approach to produce statements to develop treatment guidelines of the E&ME in RSE.

**Method:**

Experts rated their agreement with each statement on a 7‐point linear numeric scale. Consensus amongst experts was defined as a standard deviation ≤ 1. Statements attaining consensus after the first round moved to the final report. Those not attaining consensus moved to the second round in which experts were shown the mean response of the expert panel and their own response for the opportunity to reconsider their rating for that round. Statements attaining consensus after the second round moved to the final report. This process was repeated in the third round. Statements attaining consensus were moved to the final report. The remaining statements did not attain consensus.

**Results:**

Seventy‐seven experts participated in the first, 75 in the second, and 74 in the third round. Twenty‐three statements attained consensus. Twenty‐one statements did not attain consensus.

**Conclusion:**

A modified Delphi technique was used to establish consensus regarding the numerous complex factors influencing treatment of the E&ME in RSEs. Twenty‐three statements attained consensus and can be incorporated into guidelines to advance the ethical treatment of the E&ME in RSEs.

## Graphical abstract

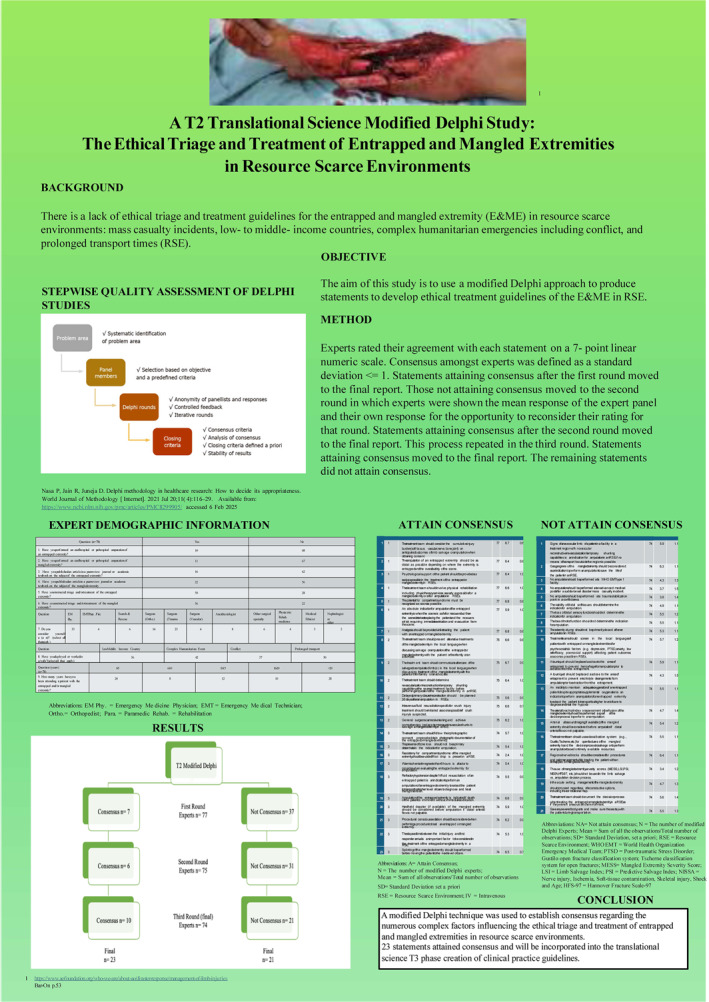

**Abbreviations**
A: attain consensus
COVID‐19: coronavirus disease 2019
CPG: clinical practice guidelines
INSARAG: world health organization and international search and rescue advisory group
IRB: institutional review board
LMIC: low‐ to low‐middle income countries
mD: modified Delphi
N: number
NA: not attain consensus
PRISMA‐ScR: preferred reporting items for systematic reviews and meta‐analyses‐scoping review extension
RSE: resource‐scarce environments
SuTra2: a prospective study of the outcome of patients with limb trauma following the Haitian earthquake in 2010 at one‐ and two‐year
T: translational science
USA: United States of America

## 1 Introduction

Emergency medical teams in resource‐scarce environments (RSE) should strive to meet the same standard of care delivered in resource‐rich environments despite a substantial difference in available resources, staff, and infrastructure [1, 2, 3, 4]. Challenges in RSE in the context after a sudden onset disaster mass casualty incident (MCI), in low‐ to low‐middle income countries (LMIC), complex humanitarian emergencies including conflict, and in settings with long transport times in remote locations have the same common paradigm shift to adapt and overcome the limitations of the supply of resources that do not meet the demands of one or more patients. The ethical triage and treatment of entrapped or mangled extremities in RSE follow a crisis standard of care framework to guide complex decisions that define immediate, short‐term, and long‐term consequences.

The SuTra2 study conducted 2 years after the 2010 earthquake in Haiti discussed the difficulties treating patients with mangled extremities with limited resources as 79% (*n* = 111) would have preferred reconstructive treatment if amputation was medically avoidable [5]. This study of patients' perceptions follows the 2011 Sonshine et al. qualitative and survey study of orthopedists that responded in Haiti reported revision surgeries at a higher rate after primary amputations, guillotine amputations, fasciotomies, and internal fixations, which in the authors' opinions were suggestive of inappropriate disaster care [6]. The authors further concluded that organizational and training barriers obstructed orthopedic care delivery immediately after the Haiti earthquake.

Clinical practice guidelines (CPG) are statements that include recommendations intended to optimize patient care that are informed by a systematic review of evidence and an assessment of the benefits and harms of alternative care options [7]. Private and public health care agencies, professional associations, health care delivery systems, or collaborations of any of these entities publish and promulgate CPGs. The issue is the influence of bias throughout CPG creation, beginning with poorly designed and executed literature reviews and the examination or testing of a CPG's efficacy, outcomes, potential for harm, and other key performance indicators.

The translational science (T) process has been adapted to disaster medicine, initiated with the clinical T0 question, “What are the ethical triage and treatment CPGs for entrapped and mangled extremities in RSEs?” [8]. This question guides the use of the preferred reporting items for systematic reviews and meta‐analyses scoping review extension [9] (PRISMA‐ScR) that searches for sources (articles) that provide themes and subthemes (data) to be incorporated into statements for the T2 modified Delphi (mD) [10]. The Delphi method is preferred as the scientific process to reach agreement amongst a group of experts on a certain issue where none previously existed to achieve the highest level of science [11]. A mD involves any variation of settings to include online (as this study did) or how the first Delphi stage statements to be presented to the Delphi experts are created.

The aim of this study was to produce final statements that attain consensus amongst a panel of experts to be utilized, incorporated, or adapted in the creation of the CPGs T3 stage. In the T4 stage, the effects and outcomes of the T3 CPGs are studied through simulations or actual incidents to be reconsidered and revised as appropriate when important new evidence warrants CPG modifications.

## 2 Materials and Methods

Two authors (EW and ZG) derived draft statements for the Delphi consensus process from three sources [12, 13, 14]. These draft statements went through an iterative review and editing process to reach consensus amongst the other authors to arrive at 44 final mD statements.

Academic researchers and educators, operational first responders, first receivers, inpatient, and outpatient clinicians treating patients with entrapped and mangled extremities in RSEs were identified to be mD experts. Email addresses were obtained from professional websites, listed on publications or collected by the authors through other collaborations. Email invitations from the lead authors (EW and ZG) to participate as an mD expert were sent over a period from June 25, 2024, through July 8, 2024. Experts that agreed were sent an email from the Stat59 (Stat59 Services Limited, Alberta, Canada) Delphi organizational program on the day that the mD began (July 8, 2024) with a link to the Stat59 website consent page. Each expert registered and validated their unique account and were sent a new email from the STAT59 platform to log into their secure webpage to begin the first mD expert consensus round.

Once the mD experts logged in, they were provided with a formal explanation of the mD methodology, and informed consent was obtained. (https://www.stat59.com/projects/delphi‐consent‐view?pid=646) Experts were notified that they were anonymous volunteers who could withdraw at any time, that participation or withdrawal would not impact their employment, and that their data were secure (https://www.stat59.com/about/security).

Only the study principal investigator (EW) and administrative author (ZG) were aware of the participating experts, though an author may have known that their colleague may have been invited to participate they did not know if they agreed to participate, nor were they privy to any of their colleague’s demographic information or responses. No expert was aware of any other expert participating in the study through study emails or the study STAT59 platform. Experts rated their agreement with each statement on a 7‐point linear numeric scale. Consensus amongst experts was defined as a standard deviation ≤ 1. Statements attaining consensus were included in the final report after the first round. Those not attaining consensus at the end of the first mD round July 23, 2024, moved to the second mD round which commenced July 23, 2024, with experts receiving a notification email from the STAT59 platform to log into the STAT59 platform using their unique username and password. Each expert was shown the mean response of the expert panel and their own response for opportunity to reconsider their rating for the statements that did not attain consensus after the first round. Experts that did not log into the STAT59 platform for round two were sent reminder emails from the author to participate in the second round.

After the second round, statements attaining consensus were included in the final report. Those not attaining consensus at the end of the second mD round, August 5, 2024, moved to the third and last mD round which commenced August 5, 2024, with experts receiving a notification email from the STAT59 platform to log into the platform using their unique username and password. Each expert was shown the mean response of the expert panel and their own response for opportunity to reconsider their rating for the statements that did not attain consensus after the second round. Experts that did not log into the STAT59 platform for round three were sent reminder emails from the author to participate in the third and last round. Statements attaining consensus after the third and final round were included in the final report. Those not attaining consensus at the end of the third mD round, August 16, 2024, were the final statements that did not attain consensus.

The University of South Florida Institutional Review Board (IRB), Tampa, Florida, United States of America (USA) determined that this protocol STUDY006895 meets the criteria for exemption from the IRB review.

## 3 Results

**FIGURE 1:**
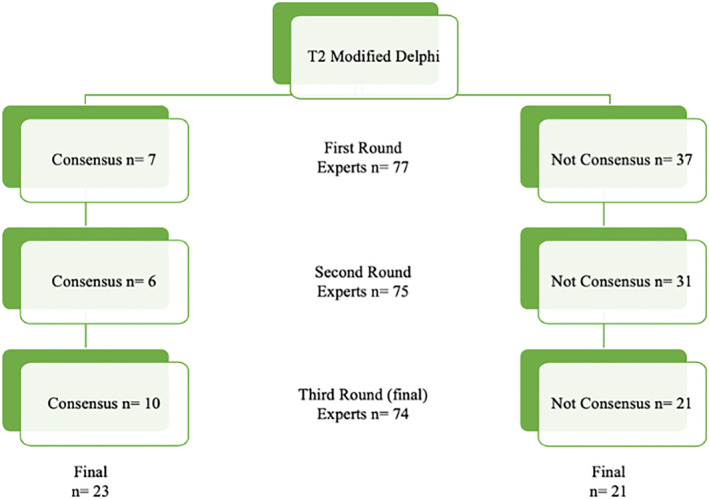
Modified Delphi results.

The results are summarized in Figure 1.

Eighty (80) mD experts confirmed their participation and established a unique account on the Stat59 website. Seventy‐nine (79) experts responded to demographic questions, 1 expert did not respond as requested, and therefore 78 experts are included in the demographic analysis. (Table 1)

**TABLE 1:** Modified Delphi expert demographic information.

Question (*n* = 78)	Yes	No
1. Have you performed an out‐of‐hospital or prehospital amputation of an entrapped extremity?	10	68
2. Have you performed an out‐of‐hospital or prehospital amputation of mangled extremity?	11	67
3. Have you published an article in a peer‐review journal or academic textbook on the subject of the entrapped extremity?	16	62
4. Have you published an article in a peer‐review journal or academic textbook on the subject of the mangled extremity	22	56
5. Have you instructed triage and/or treatment of the entrapped extremity?	50	28
6. Have you instructed triage and/or treatment of the mangled extremity?	56	22

Question	EM Phy.	EMT/Para./Fire	Search & rescue	Surgeon (ortho.)	Surgeon (trauma)	Surgeon (vascular)	Anesthesiologist	Other surgical specialty	Physiatrist Rehab. medicine	Medical ethicist	Nephrologist or other
7. Do you consider yourself a (an)? (Select all that apply)	33	4	6	14	23	4	8	6	4	3	2

Question	Low‐middle income country	Complex humanitarian event	Conflict	Prolonged transport	
8. Have you deployed or worked in a/with? (Select all that apply)	56	42	37	50	
Question (years) (*n* = 78)	0–5	6–10	11–15	16–20	> 20
9. How many years have you been attending a patient with the entrapped and/or mangled extremity?	20	8	12	10	28

Abbreviations: EM Phy. = emergency medicine physician, EMT = emergency medical technician, Ortho. = orthopedist, Para. = paramedic, Rehab. = rehabilitation.

Two (2) experts joined the panel and participated in rounds 2 and 3. One expert joined and participated in round 3 only. Three experts participated in round 1 only. One expert participated in rounds 1 and 3. (Table 2) Seventy‐seven (77) completed the first mD expert round that was open from July 8, 2024, until July 8, 2024. (Table 2)

**TABLE 2:** Modified Delphi expert participation by round.

Round	Experts lost	Experts gain	Experts in round	Total experts register
1	0	0	77	77
2	4	2	77 − 4 + 2 = 75	77 + 2 = 79
3	5	3	77 − 6 + 3 = 74	79 + 1 = 80

Seven (7) statements attained statistical significance with a standard deviation ≤ 1.0 after the first mD expert round and attained consensus. (Table 3, first round, first section in bold) The 37 statements that did not attain statistical significance, with standard deviation > 1.0, were advanced to the second mD expert round.

**TABLE 3:** Attained consensus.

A	Round	Statement	*n*	Mean	SD
1	**1**	**The treatment team should consider the cumulative injury burden (soft tissue, vascular, nerve, bone, and joint) on anticipated outcomes of limb salvage or amputation when obtaining consent.**	77	6.7	0.6
2	**1**	**The amputation of an entrapped extremity should be as distal as possible depending on where the extremity is entrapped and the overall safety of the scene.**	77	6.4	0.8
3	**1**	**Psychological support of the patient should be provided as early as possible in the treatment of the entrapped or mangled extremity in RSEs.**	77	6.4	1.0
4	**1**	**The treatment team should involve physical rehabilitation including physiotherapy services as early as possible for a mangled extremity or after amputation in RSEs.**	77	6.6	1.0
5	**1**	**The potential for compartment syndrome must be recognized as soon as possible.**	77	6.8	0.8
6	**1**	**An absolute indication for amputation of the entrapped extremity is when the scene is safe for rescuers but then the scene deteriorates placing the patient and the rescuers at risk requiring immediate extrication and evacuation from the scene.**	77	5.9	1.0
7	**1**	**Analgesia should be provided while treating the patient with an entrapped or mangled extremity.**	77	6.8	0.8
8	2	The treatment team should present alternative treatments of the mangled extremity in the local language when discussing salvage or amputation of the entrapped or mangled extremity with the patient or their family or an advocate.	75	6.6	0.8
9	2	The treatment team should communicate aftercare of the salvaged or amputated limb(s) in the local language when discussing treatment of the mangled extremity with the patient or their family or an advocate.	75	6.7	0.9
10	2	The treatment team should determine revascularization/reconstruction/temporary shunting capabilities at the nearest treatment facility before performing amputation of the mangled extremity in an RSE.	75	6.4	1.0
11	2	Delayed primary closure/reconstruction should be planned 2–5 days after an amputation in RSEs.	75	5.6	0.9
12	2	Intravenous fluid resuscitation specific for crush injury treatment should be initiated as soon as possible if crush injury is suspected.	75	6.6	0.7
13	2	General surgeons can receive training and achieve competencies to place temporary intravascular shunts to salvage a mangled extremity in a RSE.	75	6.2	1.0
14	**3**	**The treatment team should follow their photographic consent process to obtain photographic documentation of the entrapped or mangled extremity.**	74	5.7	1.0
15	**3**	**The presence of bone loss should not be a primary determinant in the indication for amputation.**	74	5.4	1.0
16	**3**	**Fasciotomy for compartment syndrome of the mangled extremity should be avoided if foot drop is present in a RSE.**	74	2.4	1.0
17	**3**	**Warm ischemia time greater than 6** **h is a factor to consider when evaluating the entrapped extremity for amputation.**	74	5.4	1.0
18	**3**	**Refractory hypotension despite IV fluid resuscitation of an entrapped patient is an indication to perform an amputation of an entrapped extremity to extract the patient to transport to a higher level of care to diagnose and treat the hypotension.**	74	5.5	0.9
19	**3**	**Amputation of the entrapped extremity is indicated if death of the patient is imminent without immediate extrication.**	74	6.6	0.9
20	**3**	**Handheld Doppler (if available) of the mangled extremity should be considered before amputation if distal arterial flow is not palpable.**	74	5.9	1.0
21	**3**	**Procedural conscious sedation should be considered when performing a procedure to treat an entrapped or mangled extremity.**	74	6.2	0.9
22	**3**	**The elapsed time between the initial injury and first responder arrival is an important factor to be considered in the treatment of the entrapped or mangled extremity in a RSE.**	74	5.3	1.0
23	**3**	**Splinting of the mangled extremity should be performed before moving the patient to the next level of care.**	74	6.5	0.7

*Note:* Rounds 1 and 3 are BOLD for emphasis.

Abbreviations: A = attain for the purpose of the discussion, IV = intravenous, Mean = sum of all the observations/total number of observations, N = the number of modified Delphi experts, RSE = resource‐scarce environment, SD = standard deviation, a measure of the amount of variation of the values of a variable about its mean set a priori.

Seventy‐five (75) mD experts completed the second mD expert round that was open from July 23, 2024, until August 5, 2024. Six (6) of the 37 statements that advanced to the second mD expert round achieved consensus (Table 3, second round middle section). The remaining 31 statements were unable to attain consensus and advanced to the third mD expert round.

Seventy‐four (74) mD experts completed the third and final mD expert round that was open from August 5, 2024 to August 16, 2024. Ten (10) of the remaining 31 statements achieved consensus; therefore, a final total of 23 statements attained consensus (Table 3, third round, last section in bold). The remaining 21 statements were unable to attain consensus after 3 mD expert rounds and were not recommended for the T3 CPG creation stage (Table 4).

**TABLE 4:** Not attain consensus.

NA	Statement	*n*	Mean	SD
1	Signs of a avascular limb of a patient in a facility in a treatment region with no vascular reconstructive/revascularization/temporary shunting capabilities are indications for amputation in an RSE if no means of transport to outside the region is possible.	74	5.9	1.1
2	Gas gangrene of the mangled extremity should be considered as an indication to perform an amputation to save the life of the patient in an RSE.	74	6.3	1.1
3	No amputation should be performed at a WHO EMT type 1 facility.	74	4.3	1.3
4	No amputation should be performed at an advanced medical post after a sudden onset disaster mass casualty incident.	74	3.7	1.5
5	No amputation should be performed at a trauma stabilization point in a conflict area.	74	3.8	1.4
6	The viability of distal soft tissues should determine the indication for amputation.	74	4.9	1.1
7	The loss of distal sensory function should not determine the indication for amputation	74	5.5	1.2
8	The loss of motor function should not determine the indication for amputation.	74	5.5	1.1
9	The extremity stump should not be primarily closed after an amputation in RSEs.	74	5.3	1.1
10	Treatment teams should screen in the local language of all patients with entrapped or mangled extremities for psychosocial risk factors (e.g., depression, PTSD, anxiety, low self‐efficacy, and poor social support) affecting patient outcomes as soon as possible in RSEs.	74	5.7	1.2
11	A tourniquet should be placed as close to the area of entrapment to prevent hemorrhage from amputation prior to extraction from the entrapment.	74	5.9	1.1
12	A tourniquet should be placed as close to the area of entrapment to prevent electrolyte derangements from amputation prior to extraction from the entrapment.	74	4.3	1.5
13	An inability to maintain adequate oxygenation of an entrapped patient despite appropriate supplemental oxygenation is an indication to perform an amputation of an entrapped extremity to extract the patient to transport to a higher level of care to diagnose and treat the hypoxia	74	5.5	1.1
14	The arterial brachial index or assessment of perfusion of the mangled extremity should be performed as part of the decision process to perform an amputation.	74	4.7	1.4
15	Arterial ultrasound imaging (if available) of the mangled extremity should be considered before amputation if the distal arterial flow is not palpable.	74	5.4	1.2
16	The treatment team should use a classification system (e.g., Gustilo and Tscherne) for open fractures of the mangled extremity to aid the decision process to salvage or to perform an amputation based on timely available resources.	74	5.5	1.1
17	Regional nerve blocks should be considered for procedures and pain management while treating the patient with an entrapped or mangled extremity.	74	6.4	1.1
18	The use of mangled extremity severity scores (MESS, LSI, PSI, NISSA, HFS‐97, etc.) should not be used in the limb salvage versus amputation decision process.	74	3.4	1.2
19	In the acute setting, management of the mangled extremity should proceed regardless of reconstructive options, including free or rotational flap.	74	4.7	1.3
20	The treatment team should document the decision process when treating the entrapped or mangled extremity in a RSE as if they were in a resource‐rich environment.	74	5.6	1.4
21	Save any severed body parts and make sure these stay with the patient during transportation.	74	5.5	1.3

Abbreviations: Gustilo = Gustilo open fracture classification system, HFS‐97 = Hannover fracture scale‐97, LSI = limb salvage index, Mean = Sum of all the observations/total number of observations, MESS = mangled extremity severity score, NISSA = nerve injury, ischemia, soft‐tissue contamination, skeletal injury, shock and age, N = the number of modified Delphi experts, NA = not attain for the purpose of the Discussion, PTSD = post‐traumatic stress disorder, PSI = predictive salvage index, RSE = resource‐scarce environment, SD = standard deviation, a measure of the amount of variation of the values of a variable about its mean set a priori, Tscherne = Tscherne classification system for open fractures, WHO EMT = world health organization emergency medical team.

## 4 Discussion

(Statements that attained consensus will be noted as A# and those that did not attain consensus as NA#.)

The adoption of CPGs to ethically triage and treat entrapped and mangled extremities in RSEs requires a trustworthy process [7] through translational science. Advances in the MCI response in a RSE is moving beyond lessons learned to a comprehensive holistic approach with an ethical core based on best available evidence and the inclusion of practitioners spanning the treatment spectrum, specifically rehabilitation [15].

The Swiss Urban Search and Rescue team followed the International Search and Rescue Advisory Group (INSARAG) guidelines to perform an amputation of a 16‐year‐old boy's right arm entrapped above the elbow 55 h after the 2023 Türkiye earthquake [16, 17]. Despite what they report as a successful amputation, utilizing conscious sedation (A21), alternative options were considered throughout the team discussion (A8) supporting the consent process of the team with the father including prompt psychological support (A3) and aftercare of the patient (A9) which were detailed in the report [17].

To aid the consent process, mD experts agreed that the treatment team should follow a photographic consent process of the entrapped extremity when discussing limb salvage or amputation (A14). This report from Türkiye provides insights into creating CPGs that explore psychological first aid for the family and members of the field treatment team [18]. Contingencies for patients and families that face the reality of surviving a sudden onset disaster with residual deficits with socioeconomic consequences and the toll of long‐term rehabilitative care in RSEs despite reasonable discussions, consent or assent process, and outcomes will have to be included in CPGs.

Consensus was attained by the mD experts that an absolute indication for the amputation of the entrapped extremity is when the scene is initially safe for the rescuers but deteriorates placing the patient and rescuers at risk (A6). Another indication to perform amputation that attained consensus is if death of the patient is imminent without immediate amputation (A19) and when there is refractory hypotension despite appropriate intravenous fluid resuscitation of the entrapped patient to transport the patient to a higher level of care to treat concomitant injuries (A18). These complex decisions will require careful consideration during the T3 stage creating CPGs.

The mangled extremity severity score [19] does include a caveat based on a 1949 study following World War II data that warm ischemia time greater than 6 h is a factor to consider in the decision process when to amputate or salvage an entrapped extremity (A17) [20]. Experts agreed that when indicated, an amputation of an entrapped extremity should be as distal as possible depending on the where the limb is entrapped and the overall safety of the scene (A2). Thus, the elapsed time between the initial injury and first responder arrival is an important factor in treatment decisions (A22). CPGs will have to include a template to capture time‐specific time points in the clinical course of the entrapped patient prior to arrival at the next level of care.

As per the standard in trauma centers, the patient with the potential for compartment syndrome must be recognized as soon as possible (A5) and intravenous fluid resuscitation specific for crush injuries should be initiated as soon as possible if crush injury is suspected (A12). Statement A16 attained consensus with a standard deviation of 1 and with a mean of 2.4. This indicated that the experts disagreed that fasciotomy for compartment syndrome should be avoided if foot drop is present in a RSE.

The administration of analgesia to the entrapped patient (A7) as well as conscious sedation (A21) for any procedures in the field should be included in CPGs. The cumulative injury burden (A1) and not the presence of bone loss (A15) in the decision process to salvage or amputate a mangled extremity attained consensus. Treatment teams should determine revascularization/reconstruction/temporary shunting capabilities (A10) of the nearest treatment facility to include local general surgeons who practice in RSEs that receive training and demonstrate competencies to place temporary vascular shunts (A13). Delayed primary closure/reconstruction should be planned 2–5 days after an amputation (A11). Handheld Doppler (if available) of the mangled extremity should be considered before amputation if distal arterial flow is not palpable (A20). Splinting of the mangled extremity moving the patient to the next level of care attained consensus (A23). The T3 CPGs will require initial asynchronous and synchronous blended simulation education and training to achieve competencies and then regular maintenance programs to maintain competencies to perform these procedures in challenging RSE.

To maximize the return of the patient to their highest level of function, appropriate prosthetics and orthoses in a RSE with limited rehabilitation staff, stuff, and structures (staffed clinics and means of transportation of the patient to the facility) are paramount as these clearly factor into treatment decisions. The planned T3 stage of this study will include the involvement of physiatrists, as early as possible, who are familiar with the challenges of rehabilitation in RSEs to guide initial pre‐hospital, advanced medical post or trauma stabilization point next echelon care prior to definitive care (A4).

The documentation of warm ischemia time (A17) and elapsed time (A22) description of the cumulative burden of injuries (A1) is an important factor in treatment decisions of the entrapped and mangled extremity. Documentation of the communication with the patient, their family, or representative (A8‐9) is no different than the standard practice in noncrisis settings. However, mD experts did not agree that the treatment team should document the decision process when treating the entrapped or mangled extremity (NA20). This incongruity requires further analysis to achieve the standard of documentation of the clinical course.

**FIGURE 2:**
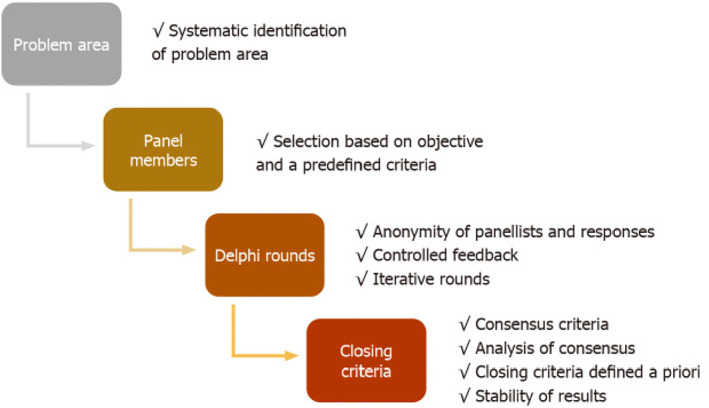
Stepwise quality assessment of Delphi studies.

The science supporting the use of the mD to obtain consensus where consensus did not exist previously has become more salient recently. This study followed the use of the 1–7 linear numeric scale, the standard deviation, the mean to analyze mD data suggested by Franc et al. [21], and the stepwise quality assessment detailed by Nasa et al. [22] (Figure 2) This does not elevate consensus from the lowest level of evidence but can provide a measure of security in the methodology to maximize the quality of the data produced.

## 5 Conclusions

The statements presented to 80 experts through three mD rounds for the purpose of this translational science project follow the 4 themes: ethics, triage, treatment, and rehabilitation. The T3 stage will create CPGs based on the 23 statements that attained consensus, treating these 4 themes not as separate entities but as a holistic effort to be adapted to the circumstances of the injury in the setting of the RSE.

## Author Contributions

**Eric S. Weinstein:** conceptualization, data curation, formal analysis, investigation, methodology, project administration, software, supervision, validation, visualization, writing‐original draft, writing‐review and editing. **Zachary Gilbert:** data curation, formal analysis, investigation, methodology, project administration, software, writing‐review and editing. **James Gosney:** conceptualization, formal analysis, investigation, methodology, validation, visualization, writing‐review and editing. **Brielle Weinstein:** conceptualization, methodology, validation, visualization, writing‐review and editing. **Hannah B. Wild:** conceptualization, data curation, validation, visualization, methodology, writing‐review and editing. **Joseph Cuthbertson:** conceptualization, methodology, validation, visualization, writing‐review and editing. **Melissa Leming:** conceptualization, validation, visualization, methodology, writing‐review and editing. **Rachel Semmons:** conceptualization, validation, visualization, methodology, writing‐review and editing. **Dónal O'Mathúna:** conceptualization, validation, visualization, methodology, writing‐review and editing. **Carl Montan:** conceptualization, validation, visualization, methodology, writing‐review and editing. **Richard Gosselin:** conceptualization, validation, visualization, methodology, writing‐review and editing. **Frederick “Skip” Burkle:** conceptualization, investigation, methodology, supervision, validation, visualization, writing‐review and editing.

## Ethics Statement

The University of South Florida Institutional Review Board (IRB), Tampa, Florida, United States of America (USA) determined that this protocol STUDY006895 meets the criteria for exemption from the IRB review.

## Consent

Each modified Delphi expert provided consent.

## Conflicts of Interest

The authors declare no conflicts of interest.

## Supporting information

Table S1

## References

[1] D. Hanfling , J. L. Hick , and C. Stroud , “Committee on Crisis Standards of Care: A Toolkit for Indicators and Triggers; Board on Health Sciences Policy,” in Institute of Medicine. Crisis Standards of Care: A Toolkit for Indicators and Triggers (Washington, DC: National Academies Press [US], Sep, 2013). PMID: 24872980. 24872980

[2] A. Kaji , B. Hansoti , and M. Boukhman . 2013. “Guidelines for Crisis Standards of Care During Disasters,” https://www.acep.org/globalassets/uploads/uploaded‐files/acep/clinical‐and‐practice‐management/policy‐statements/information‐papers/guidelines‐for‐crisis‐standards‐of‐care‐during‐disasters.pdf.

[3] World Health Organization . 2021. “Refugee and Migrant Health Global Competency Standards for Health Workers,” https://www.who.int/publications/i/item/9789240030626.

[4] UN. Committee on Economic, Social and Cultural Rights. 41st sess . 2008. “Substantive Issues Arising in the Implementation of the International Covenant on Economic, Social and Cultural Rights. Committee on Economic, Social and Cultural Rights,” in International Standards on the Right to Physical and Mental Health. General Comment No. 14 (2000).The Right to the Highest Attainable Standard of Health (Article 12 of the International Covenant on Economic, Social and Cultural Rights), https://digitallibrary.un.org/record/647781?ln=en.

[5] M. C. Delauche , N. Blackwell , H. Le Perff , et al., “A Prospective Study of the Outcome of Patients With Limb Trauma Following the Haitian Earthquake in 2010 at One‐ and Two‐ Year (The Sutra2 Study),” PLOS Currents 5 (July 5 2013): 5–ecurrents, 10.1371/currents.dis.931c4ba8e64a95907f16173603abb52f. PMC4011624 24818064

[6] D. B. Sonshine , A. Caldwell , R. A. Gosselin , C. T. Born , and R. R. Coughlin , “Critically Assessing the Haiti Earthquake Response and the Barriers to Quality Orthopaedic Care,” Clinical Orthopaedics and Related Research 470, no. 10 (2012): 2895–2904, 10.1007/s11999-012-2333-4. 22487879 PMC3442014

[7] R. Graham , M. Mancher , and D. M. Wolman , “Clinical Practice Guidelines We Can Trust,” in Institute of Medicine (U.S.). Committee on Standards for Developing Trustworthy Clinical Practice Guidelines (Washington, DC: National Academies Press, 2011). ISBN‐13: 978‐0‐309‐16422‐1ISBN‐13: 978‐0‐309‐16423‐8.

[8] National Center for Advancing Translational Sciences . 2024. “About Translational Science,” in NIH National Center for Advancing Translational Science, https://ncats.nih.gov/about/about‐translational‐science.

[9] A. C. Tricco , E. Lillie , W. Zarin , et al., “PRISMA Extension for Scoping Reviews (PRISMA‐ScR): Checklist and Explanation,” Annals of Internal Medicine 169, no. 7 (2018): 467–473, 10.7326/m18-0850. 30178033

[10] E. S. Weinstein , J. L. Cuthbertson , L. Ragazzoni , and M. Verde , “A T2 Translational Science Modified Delphi Study: Spinal Motion Restriction in a Resource‐Scarce Environment,” Prehospital and Disaster Medicine 35, no. 5 (2020): 538–545, 10.1017/s1049023x20000862. 32641192

[11] H. McKENNA , S. Keeney , and F. Hasson , “Health Care Managers’ Perspectives on New Nursing and Midwifery Roles: Perceived Impact on Patient Care and Cost Effectiveness,” Journal of Nursing Management 17, no. 5 (2009): 627–635, 10.1111/j.1365-2834.2008.00948.x. 19575721

[12] E. Weinstein , J. E. Gosney , L. Ragazzoni , et al., “The Ethical Triage and Management Guidelines of the Entrapped and Mangled Extremity in Resource Scarce Environments: A Systematic Literature Review,” Disaster Medicine and Public Health Preparedness 15, no. 3 (2020): 389–397, 10.1017/dmp.2020.49. 32456743

[13] AO Foundation . 2016. “A Field Guide—Management of Limb Injuries,” https://www.aofoundation.org/what‐we‐do/innovation‐translation/innovation‐funding/portfolio/a‐field‐guide.

[14] C. B. K. Potter and M. J. Bosse , “American Academy of Orthopaedic Surgeons Clinical Practice Guideline Summary for Limb Salvage or Early Amputation,” Journal of the American Academy of Orthopaedic Surgeons 29, no. 13 (2021): e628–e634, 10.5435/jaaos-d-20-00188. 33878076

[15] K. L. Koenig , C. H. Schultz , M. Gould Runnerstrom , and O. A. Ogunseitan , “Public Health and Disasters: An Emerging Translational and Implementation Science, Not ‘Lessons Learned’,” Disaster Medicine and Public Health Preparedness 11, no. 5 (2017): 610–611, 10.1017/dmp.2017.11. 28330523

[16] United Nations Office for the Coordination of Humanitarians Affairs (OCHA) . 2020. “INSARAG Guidelines,” https://www.insarag.org/methodology/insarag‐guidelines/.

[17] M. Franz , D. Lionel Bernard , M. Mona , J. Jean‐Daniel , B. Urs , and H. Olivier , “Life‐saving Field Amputation During the 2023 Türkiye Earthquake: Ethical, Social, and Legal Implications Beyond the Complex Medical and Rescue Procedures in the Rubble,” Disaster Medicine and Public Health Preparedness 18 (2024): e131, 10.1017/dmp.2024.110. 39291327

[18] L. Snider , A. Schafer , M. van Ommeren , et al., Psychological First Aid: Facilitator’s Manual for Orienting Field Workers (WHO Press, World Health Organization, 2013), https://www.who.int/publications/i/item/psychological‐first‐aid.

[19] K. Johansen , M. Daines , T. Howey , D. Helfet , and S. T. Hansen Jr. , “Objective Criteria Accurately Predict Amputation Following Lower Extremity Trauma,” Journal of Trauma, Injury, Infection, and Critical Care 30, no. 5 (1990): 568–573, 10.1097/00005373-199005000-00007. 2342140

[20] H. H. Miller and C. S. Welch , “Quantitative Studies on the Time Factor in Arterial Injuries,” Annals of Surgery 130, no. 3 (1949): 318–438, 10.1097/00000658-194909000-00010. PMC1616504 17859442

[21] J. Franc , K. Hung , A. Pirisi , and E. Weinstein , “Analysis of Delphi Study Seven‐Point Linear Scale Data by Parametric Methods–Use of the Mean and Standard Deviation,” supplement, Prehospital and Disaster Medicine 38, no. S1 (2023): s30–s31, 10.1017/s1049023x23001188.

[22] P. Nasa , R. Jain , and D. Juneja , “Delphi Methodology in Healthcare Research: How to Decide its Appropriateness,” World Journal of Methodology 11, no. 4 (2021): 116–129, https://www.ncbi.nlm.nih.gov/pmc/articles/PMC8299905/. 34322364 10.5662/wjm.v11.i4.116 PMC8299905

