# Supplementary material for: A T2 Translational Science Modified Delphi Study: The Ethical Triage and Treatment of Entrapped and Mangled Extremities in Resource‐Scarce Environments
Source: World J Surg. 2025 Feb 20;49(4):1051–60. doi: 10.1002/wjs.12486 (PMC11994154; doi:10.1002/wjs.12486)
Supplement: Supplementary file 1 — Table S1 [file WJS-49-1051-s001.pdf]

TABLE S1 ACKNOWLEDGEMENT OF MODIFIED DELPHI EXPERTS

Mohana	Amirtharajah	Alba	Ripoll Gallardo	Nelson	Olim
Laura	Archer	Daniele	Gui	Ahmad	Oqlat
Bracken	Armstrong	Pinchas	Halpern	Nia	Owens
Elhanan	Bar-On	Ahmad	Alchaikh Hassan	Ronald	Pirrallo
Michael	Bentele	Henrik	Hedelin	Arul	Ramasamy
Giacomo	Biagetti	Mariana	Helou	Farooq	Rathore
Michael	Bosse	Joe	Holley	Amila	Ratnayake
Valeria	Caramello	Chi-Feng	Hsu	Yohan	Robinson
Renan	Castillo	Angus	Jameson	Madeline	Ross
Stanley	Chartoff	Henrik	Jörnvall	S Raja	Sabapathy
James	Clover	Andy	Kent	Sushma	Sagar
Michael	Court	Anna	Kerola	Andrew	Schmidt
Phil	Cowburn	Yoram	Klein	Richard	Schwartz
Dulantha	De Silva	Knut Magne	Kolstadbråten	Mehmet	Sever
Bahtiyar	Demiralp	Maria	Lampi	Wayne	Smith
Kenneth	Dumas	Nathan	Liang	Barclay	Stewart
Khalid	Eddahiri	Luis	Llerena	Riccardo	Stucchi
Roberto	Faccincani	Michael	Lozano	Johannes	Svensøy
James	Flippin	Ilaria	Morelli	Jonathan	Tilsed
Anthony	Fong	Ali	Mulla	Jennifer	Verstreken
Joseph	Forrester	Erisa	Mwaka	George	Voicescu
Francesco	Foti	Michael	Mwandri	Sherry	Wren
Pietro	Fransvea	Nicholas	Namias	Carlos	Yánez
		Colleen	O'Connell	Yavuz	Yiğit

We thank each expert that consented to be acknowledged the others who did not consent without your time, expertise and support of the project we could not have been successful.
